# New insight into ectopic thyroid glands between the neck and maxillofacial region from a 42-case study

**DOI:** 10.1186/s12902-015-0066-6

**Published:** 2015-11-18

**Authors:** Ting Gu, Boren Jiang, Ningjian Wang, Fangzhen Xia, Lizhen Wang, Aichun Gu, Feng Xu, Yongshun Han, Qin Li, Yingli Lu

**Affiliations:** Institute and Department of Endocrinology and Metabolism, Shanghai Ninth People’s Hospital, Shanghai JiaoTong University School of Medicine, Shanghai, 200011 China; Department of Oral Pathology, Shanghai Ninth People’s Hospital Affiliated to Shanghai JiaoTong University School of Medicine, Shanghai, China; Department of Nuclear Medicine, Shanghai Ninth People’s Hospital Affiliated to Shanghai JiaoTong University School of Medicine, Shanghai, China; Department of Radiology, Shanghai Ninth People’s Hospital Affiliated to Shanghai JiaoTong University School of Medicine, Shanghai, China

**Keywords:** Ectopic thyroid, Lingual thyroid, Thyroid transcription factor-1, Calcitonin, Malignant cells

## Abstract

**Background:**

Ectopic thyroid is a rare disease. In the present study at the 9th People’s Hospital in Shanghai, China, 42 patients’ ectopic thyroid glands between the neck and maxillofacial region were subjected to a retrospective and transverse study based on data from 1978 to 2012 to explore the natural characteristics of ectopic thyroid.

**Methods:**

The patients’ clinical data were collected. In addition, scintigraphy (Tc-99 m, Iodine-131), CT scan, histology and pathology were performed. The protein expression of thyroid transcription factor-1 (TTF-1), thyroglobulin (TG), calcitonin (CT), Ki-67 and parathyroid hormone (PTH) were analyzed from paraffin wax-stored specimens of ectopic thyroid tissue compared with those of orthotopic thyroid tissue.

**Results:**

There were 42 total ectopic thyroid patients, approximately 1.24 patients per year on average at our hospital. These patients were aged from 6 to 85 years old, and there were 35 females (83.3 %), seven males (16.7 %). In total, 27 of the patients had lingual thyroid (64 %); seven, sublingual thyroid (17 %); five, dual areas occupied by ectopic thyroid (12 %) and three, other types (7 %). The following conditions were also presented: nodular goiter (13 %), adenoma (8.7 %) and Hashimoto’s thyroiditis (4.3 %), no malignancy and no accompanying ectopic parathyroid. TTF-1 expression was significantly higher in ectopic samples than that in orthotopic samples (*P* = 0.007), but CT and Ki-67 levels displayed no difference. PTH was negative in ectopic tissue.

**Conclusion:**

Ectopic thyroid is a rare disease and females were more prone to the disease. The most frequent location was lingual thyroid. Nodular goiter, adenoma and Hashimoto’s thyroiditis was observed as orthotopic thyroid without accompanying ectopic parathyroid. TTF-1 was highly expressed in ectopic tissue, which may be related to abnormal embryogenesis leading to the thyroid gland being in an abnormal position. The expression of calcitonin (CT) and Ki-67 was not increased, and there were no malignant cells in any sample, which could indicate that it is not easy for ectopic thyroids to become malignant between the neck and maxillofacial region.

## Background

Ectopic thyroid refers to thyroid tissue being in locations other than the normal anterior neck region between the second and fourth tracheal cartilages. It is a rare developmental abnormality, resulting from aberrant embryogenesis of the thyroid gland during its passage from the floor of the primitive foregut to its final pre-tracheal position [1, 2]. The prevalence of ectopic thyroid is approximately 1 per 100,000–300,000 people [3]. One major clinical concern regarding ectopic thyroid glands is their potential risk for malignant transformation [4, 5]. Functional abnormalities associated with ectopic thyroid have also been well recognized. Individuals with ectopic thyroid often suffer hypothyroidism. A number of case reports of ectopic thyroid have been reported. However, no systematic analysis on protein expression of key thyroid factors using a large number of ectopic thyroid tissues has been reported. In this study, we chose several important factors including thyroid transcription factor-1, thyroglobulin, Ki-67, calcitonin, and parathyroid hormone as study target to explore the functional abnormalities that may be associated with the ectopic thyroid glands.

Thyroid transcription factor-1 (TTF-1) and thyroglobulin (TG) are important protein markers for thyroid gland function. TTF-1, also known as Nkx2.1 or thyroid-specific enhancer binding-protein, is a 38-kDa DNA-binding protein containing 371 amino acids. It is encoded by a gene located on chromosome 14q13 and is preferentially expressed in the thyroid and lungs [6]. In the thyroid, TTF-1 is expressed in follicular cells and C-cells in developing and adult thyroid glands, where it activates thyroglobulin and thyroperoxidase gene transcription [7]. However, it is not clear if alterations in TTF-1 might be involved in the ectopic thyroid.

Located on a chromosome 8q24, TG gene consists of 48 exons and encodes a protein with 2768 amino acids [8]. TG is exclusively synthesized in the thyroid gland and represents a highly specialized homodimeric glycoprotein for thyroid hormone biosynthesis [9].

Ki-67 is a large nucleolar phosphoprotein that participates the regulation of cell cycle and cell proliferation. Detailed cell cycle analysis revealed that Ki-67 antigen is present in the nuclei of actively proliferating cells (G1, S and G2 phase and mitosis) but not in the nuclei of quiescent or resting cells (G0 phase) [10]. The fraction of Ki-67-positive tumor cells (the Ki-67 labeling index) is often correlated with the clinical course of cancer patients [11]. Calcitonin (CT) is a 32-amino acid polypeptide hormone secreted from parafollicular cells of the thyroid gland [12]. Calcitonin is high expressed in medullar thyroid cancer tissues [13]. With these considerations, we compared the expression levels of Ki-67 and CT protein in ectopic thyroid tissues with orthotopic thyroid gland to evaluate the risk for malignant transformation.

Generally, orthotopic thyroid is usually accompanied by 4 parathyroid glands. It remains unclear if ectopic parathyroid gland exist with ectopic thyroid and if parathyroid functional abnormalities may be present in such individuals. Parathyroid hormone (PTH) is produced by parathyroid gland and is considered an indicator of parathyroid gland function. In this study, we measured the expression of PTH in ectopic tissue as a marker for parathyroid gland.

The Shanghai Ninth People’s Hospital Affiliated to Shanghai JiaoTong University School of Medicine is well-known for its expertise in head and neck maxillofacial surgery involving stomatology. For the past 34 years, this hospital has accumulated relatively large cases of ectopic thyroid. The ample source of cases has provided a unique opportunity to investigate the clinical manifestation as well as morphological and functional abnormalities related to ectopic thyroid glands.

## Methods

### Study design and participants

Ectopic thyroid patients were identified, during 1978 to 2012, from the medical records of hospitalized and outpatient patients. The clinical data including age, sex, symptom, thyroid function, imaging (① ultrasonography, ② CT or MRI scan, ③ 131 Iodine, ④ 99mTc-pertechnetate), laryngofiberscopy, FNAC (fine needle aspiration cytology) and operation condition, were collected and analyzed. This project was approved by the Institutional Review Board (IRB) of the Shanghai Ninth People’s Hospital, which is affiliated with the Shanghai Jiaotong University School of Medicine. All participants received oral and written information and gave written consent to participate.

### Immunohistochemistry

Pathological specimens were obtained from the pathology library. Twenty-three ectopic thyroid patients (16 patients with lingual thyroid, five with sublingual-type thyroid and two with dual ectopic thyroid) among the 42 patients underwent surgical excision. Since 2 pathologic specimens (one lingual thyroid and one sublingual thyroid) were missing, 21 paraffin fixed specimens are available for histological study. 7 were males and 14 were females, at ages of 37 to 70 years old (51.95 ± 8.69). 21 orthotopic thyroid samples were included as controls. Normal thyroid tissues were collected from patients underwent surgery for nodular thyroid or thyroid adenoma. We also collected specimens from one papillary thyroid carcinoma, one medullary thyroid carcinoma and one parathyroid gland for comparison.

The following antibodies were used for immunohistochemostry: Monoclonal Mouse Anti–human TTF-1 (clone 8G7G3/1, Dako) at a dilution of 1:50, Monoclonal Mouse Anti–human Thyroglobulin (clone DAK-Tg6, Dako) at a dilution of 1:100, Monoclonal Rabbit Anti–human Calcitonin (clone SP17, Dako) at a dilution of 1:100, Monoclonal Mouse Anti–human Parathyroid Hormone (clone 105G7, Dako) at a dilution of 1:150 and Monoclonal Mouse Anti–human Ki-67 (clone MIB-1, Dako) at a dilution of 1:75. EnVisionTM HRP (horseradish peroxidase) RABBIT/MOUSE (K5007, Dako) was also used.

Immunohistochemical staining was performed with a two-step EnVision method. All tissue biopsies were fixed in 10 % buffered formalin, embedded in paraffin and cut into 5-μm sections. After deparaffinization, heat-induced epitope retrieval was performed for 20 min using Tris-EDTA PH 9.0 for TTF-1, CT and Ki-67, and 10 mmol/L citrate buffer PH 6.0 for TG and PTH. The samples were then cooled, washed and placed in 3 % hydrogen peroxide for 10 min. After 60 min of incubation with primary antibody and 30 min of incubation with secondary antibody, color development was performed with DAB+ reagents (Dako). Slides were counterstained with hematoxylin and mounted for microscopic observation.

Semi-quantitative analysis of staining intensity was carried out with the use of Image-Pro-Plus 6.0. TG levels were determined by mean optical density (MOD), which was calculated by integrated optical density (IOD)/area of positive-staining. Nuclear-expressed proteins, such as TTF-1 and Ki-67, were evaluated by the percentage of positive cells, which was calculated by the number of positive cells/number of total cells multiply by 100. We took three 400-fold-photos for each case for statistical analysis.

### Statistics

The data are presented as the mean ± SD. The Student *t* test was used to analyze mean differences between the ectopic and orthotopic groups, and *P* < 0.05 was considered statistically significant.

## Results

### Clinical manifestations

Forty-two patients came to this hospital, with approximately 1.24 people per year among the whole patient population, including 16,181 ± 9953 inpatients/year and 1,780,429 ± 193,641 outpatients/year. The patients with ectopic thyroid were aged between 6 and 85 years. There were 35 females (83.3 %) and 7 males (16.7 %). Their symptoms included sensation of a foreign body, dysphagia, dyspnea, pain, dysphonia, snore, hemorrhage and cough. Seventeen cases appeared to be asymptomatic (Tables 1 and 2).

**Table 1:** Demographic information of 42 ectopic thyroid cases

Clinical values	Cases	Percentage
Sex		
Male	7	16.7 %
Female	35	83.3 %
Age		
< 10 years	2	4.8 %
10–19 years	6	14.3 %
20–29 years	5	11.9 %
30–39 years	10	23.8 %
40–49 years	9	21.4 %
> =50 years	10	23.8 %

**Table 2:** Clinical manifestations and thyroid function of 42 ectopic thyroid cases

Manifestation	Cases	Thyroid function	No. (%) with data
Asymptomatic	17	Euthyroidism	47.0 %
Sensation of foreign body	16	Hypothyroidism	5.9 %
Dysphagia/dyspnea	5/5	Subclinical hypothyroidism	35.3 %
Pain/dysphonia	4/3	Subclinical hyperthyroidism	5.9 %
Snoring/hemorrhage/cough	2/1/1	Others	5.9 %

### Thyroid function

Thyroid function was normal for 8 cases. There was 1 case with hypothyroidism: TSH 34.85 uIu/L (0.34–5.6) (↑), FT3 3.53 pg/ml (1.71–3.71), FT4 0.53 ng/dl (0.7–1.48) (↓), TT3 1.13 ng/ml (0.58–1.59), TT4 5.86 ug/dl (4.8–11.58). There were 6 cases of subclinical hypothyroidism (with elevated TSH, FT3, FT4, TT3 and TT4 were normal). There was one case of subclinical hyperthyroidism: TSH 0.005 uIu/L (0.34–5.6) (↓), and FT3, FT4, TT3 and TT4 were normal. There was 1 other case: TSH 4.68 uIu/L (0.34–5.6), FT3 4.10 pg/ml (1.71–3.71) (↑), FT4 1.88 ng/dl (0.7–1.48) (↑), TT3 0.78 ng/ml (0.58–1.59) and TT4 12.38 ug/dl (4.8–11.58) (↑). In total, there were eight euthyroidism (47.0 %) cases, seven cases (41.2 %) of hypothyroidism including subclinical hypothyroidism, one case (5.9 %) of subclinical hyperthyroidism and one (5.9 %) other case (Table 2).

### Imaging examination

Twenty-two patients underwent ^131^Iodine scan. Sixteen patients accepted 99mTc-pertechnetate scan. Nineteen patients underwent CT scan, and six patients received an MRI. Laryngofiberscopy was used for four patients. Ultrasonography was used for 18 patients, and tissue biopsy or FNAC were used for seven patients (Table 3).

**Table 3:** The method of diagnosis and imaging of 42 ectopic thyroid cases

Examination	Cases
Ultrasonography	18
CT	19
MRI	6
^131^Iodine	22
99mTc-pertechnetate	16
Laryngo-fiberscope	4
Tissue biopsy or FNAC	7

### Anatomical location

**Fig. 1:**
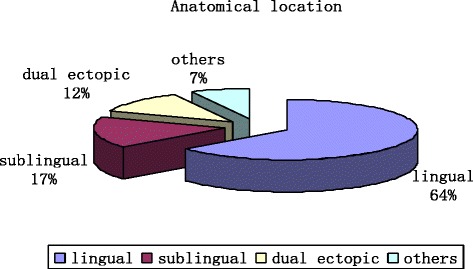
Anatomical location of 42 ectopic thyroids. Twenty-seven patients with lingual thyroid (64 %), 7 patient with sublingual types (17 %), 5 patients with dual ectopic thyroid (12 %) and 3 patients with other types (7 %)

**Fig. 2:**
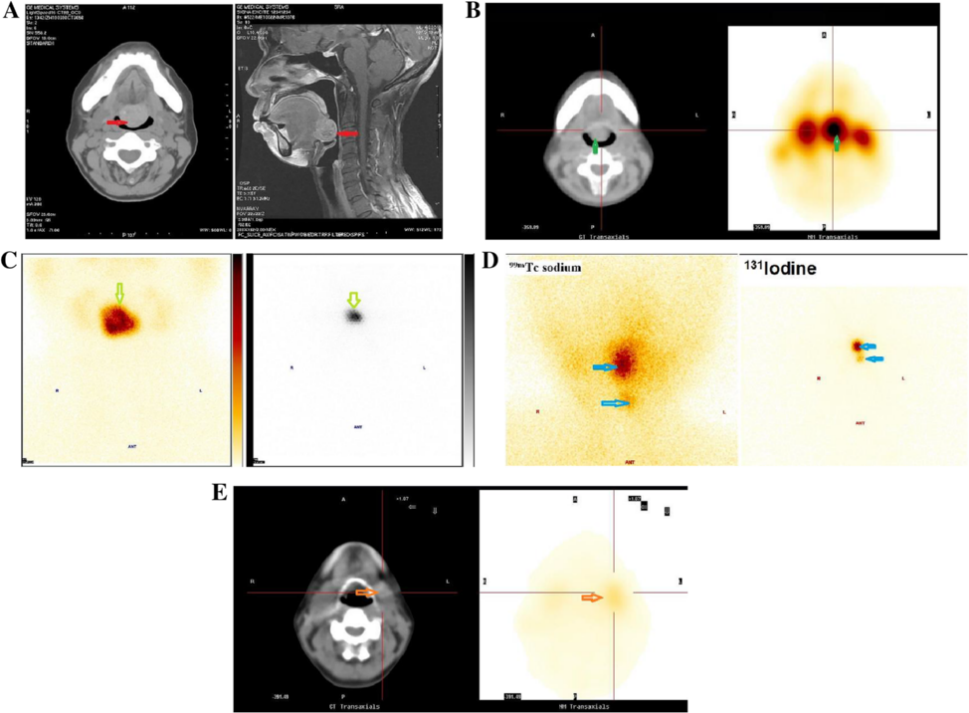
**a** Lingual thyroid in CT scan and MRI. A 55-year-old woman with lingual thyroid sufferred from sensation of a foreign body in the throat. Nonenhanced axial CT (*left*), and contrast-enhanced sagittal MRI (*right*) images showing a mass (*red arrow*) at the base of the tongue. **b** Lingual thyroid in SPECT/CT. A technetium-99 thyroid scan shows lingual thyroid as indicated by the green arrow. This is the same patient as Fig. 2a. **c** Sublingual thyroid detected with 99mTc-pertechnetate and ^131^Iodine. A 48-year-old man with sublingual thyroid. A technetium-99 thyroid scan (*left*) and ^131^I scan (*right*) showing marked uptake in submental area (*arrow*) and no uptake in the neck. **d** Dual ectopic thyroid in ^99m^Tc sodium and ^131^Iodine images. This was a 6-year-old female patient. Technetium-99 m pertechnetate and ^131^iodine imaging revealed two ectopic foci in the lingual and submental areas simultaneously, and no orthotopic thyroid was found. **e** Other type of ectopic thyroid in CT and ECT. SPECT/CT imaging with ^99m^TcO4^−^ reveals ectopic thyroid as indicated by arrows in the left submandibular area. This patient was a 38-year-old female. The FNAC indicated a small amount of thyroid tissues

As shown in Fig. 1, twenty-seven patients had a lingual thyroid (64 %), and seven patients had sublingual-type thyroids (17 %). Five patients presented dual ectopic thyroid (12 %), and three patients had other types (7 %). In all five dual ectopic cases, the first lesion was lingual and the second was sublingual (one in the floor of the mouth, and four in anterior neck region). In three other type cases, the ectopic thyroids located substernal. Ten patients had orthotopic thyroid but still had thyroid tissue in other area (23.8 %). The representative images from the CT scan, MRI, 99mTc-pertechnetate and ^131^Iodine are shown in Fig. 2.

### Pathologic assessment

**Fig. 3:**
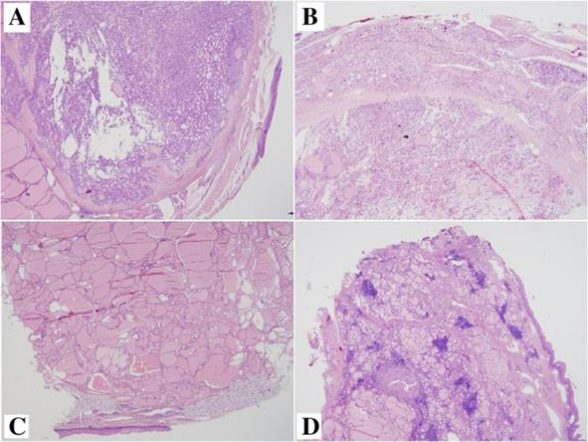
Different histology types of ectopic thyroid. Histological images of different benign conditions found in ectopic thyroid tissues. **a** adenomatous hyperplasia; **b** multiple nodular goiter; **c** colloid goiter; **d** Hashimoto’s thyroiditis (HE, ×20)

Ectopic thyroid was found to be smaller than that of orthotopic thyroid. Three cases presented nodular goiters. Two cases had thyroid adenoma. One case presented colloid goiter. One case presented thyroid cysts. One case had Hashimoto’s thyroiditis, and 15 cases were normal thyroid tissues. No malignant cells and no accompanying ectopic parathyroid glands were found in all patients (Fig. 3).

### Immunohistochemistry results

**Fig. 4:**
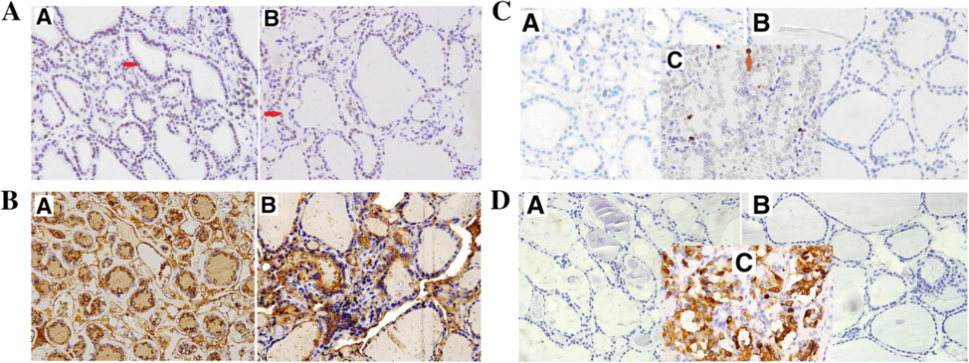
**a** TTF-1 immunostaining of the thyroid. Positive TTF-1 staining was found in the nucleus of follicular cells in both (*a*) ectopic and (*b*) orthotopic thyroids. Positive cells in brown color were indicated by red arrow. TTF-1 expression was significantly higher in the ectopic thyroids than orthotopic thyroids (×400). The quantification data was shown in Table 4. **b** Thyroglobulin (TG) expression in thyroid. *a* ectopic thyroid. *b* orthotopic thyroid (×400). Positive staining (*brown color*) of thyroglobulin was found in the cytoplasm of thyroid follicular cells and extracellular areas in both ectopic and orthotopic thyroids. There were no significant differences between the ectopic and othortopic thyroid tissues. **c** Immunostaining of Ki-67 in the thyroid. *a* ectopic thyroid. *b* orthotopic thyroid. *c* Papillary thyroid carcinoma. (×400). In both the ectopic and orthotopic thyroids, the expression of Ki-67 was very low in thyroid follicular cells. Strong staining signals for Ki-67, as indicated by the arrow, was found in the papillary thyroid carcinoma. **d** Immunostaining of calcitonin in the thyroid. *a* ectopic thyroid. *b* orthotopic thyroid. *c* The medullary thyroid carcinoma. The ectopic thyroid and orthotopic thyroids were negative for calcitonin expression. The medullary thyroid carcinoma of thyroid showed a strongly positive staining for calcitonin

TTF-1 is expressed in follicular cells. However, a significantly increased expression of TTF-1 was observed in ectopic group in comparison to orthotopic group (56.7 ± 14.8 vs 49.1 ± 13.9, respectively, *P* = 0.007). Thyroglobulin (TG) was positive in thyroid follicular cells for all cases examined and no significant difference was detected between the ectopic and control groups (0.1265 ± 0.0252 vs 0.1295 ± 0.0241, respectively, *P* = 0.6636) (Fig. 4a, b and Table 4).

**Table 4:** The expression levels of TTF-1 and TG

Group	TTF-1	TG
Ectopic thyroid (n = 21)	56.7 ± 14.8	0.1265 ± 0.0252
Orthotopic thyroid (n = 21)	49.1 ± 13.9	0.1295 ± 0.0241
*P value	0.007	0.6636

*represents comparison between ectopic thyroid and orthotopic thyroid groups

Low levels of staining signals of Ki-67 was detected in thyroid follicular cells from both ectopic and orthotopic thyroids. In addition, both ectopic and orthotopic thyroids displayed much lower levels of Ki-67 expression when compared to papillary thyroid carcinoma tissues (Fig. 4c).

Calcitonin staining was negative in parafollicular cells in all ectopic thyroid tissue. Three cases were found to be weakly positive with orthotopic thyroids. As a positive control, the medullary thyroid carcinoma displayed strongly positive staining for calcitonin (Fig. 4d).

Immunostaining of parathyroid hormone (PTH) was negative in ectopic tissues and orthotopic thyroids. As a positive control, a normal parathyroid gland showed strong staining.

The immunohistochemistry results showed significantly higher TTF-1 protein levels in ectopic thyroid tissues than orthotopic tissues.

## Discussion

Lingual thyroid is still a rare clinical entity. This developmental anomaly is the result of an arrested descent of the gland anlage early in the course of embryogenesis [14]. To the best of our knowledge, this current study is one of the largest case series concerning ectopic thyroid reported up to now [3, 15]. Clinical symptoms are typically related to the size and location as well as thyroid function. In this study, some patients presented symptoms such as the sensation of a foreign body. However, there were 40.5 % of patients appeared to be asymptotic. Insignificant symptoms were easily missed and in some cases the symptoms were retrospectively recalled during treatment of other diseases.

It is generally accepted that lingual thyroid is the most frequent ectopic location, accounting for about 90 % of the reported cases, although lower rates (47 %) have also been reported by others [2, 3, 15]. Our data indicated that lingual thyroid is the most common type, accounting for 64 %. Females are the predominant suffers of this disease. We suspect that females may be vulnerable to certain embryonic mutations affecting the development between the second and fourth tracheal cartilages. Genetic studies have demonstrated that transcription factors TITF-1 (Nkx2-1), Foxe1 (TITF-2) and PAX-8 may be involved in the abnormal migration of the thyroid [16, 17]. Further study is needed to investigate the associated genes in females with ectopic thyroid.

Radioisotope imagining was the most used form of imaging in our study. 99mTc or ^131^Iodine imaging often delivers important diagnostic information for the presence of ectopic thyroid tissue. Technetium-99 pertechnetate yields better quality imaging and imposes lower radiation burden to the body compared to iodine-131, which has been frequently applied in thyroid medicine for the past two decades. However, it accumulates in the salivary glands, making it difficult to distinguish small masses. Therefore, ^131^Iodine procedure is still required for a definitive diagnosis. CT scans and MRI are valuable tools in identifying the site of ectopy, especially when it is distant from the descending pathway of the thyroid. Tissue biopsy for histology or fine needle aspiration cytology (FNAC) provides considerable assistance in confirming the diagnosis of ectopic thyroid. They are especially useful methods for differentiation diagnosis between benign and malignant lesions. However, FNAC results may sometimes be misleading or non-diagnostic, especially for cystic masses.

In the present study, we found that nodular goiter, adenoma, Hashimoto’s thyroiditis, colloid goiter and thyroid cyst might occur in ectopic thyroid as well as in orthotopic thyroid glands. These lesions may be related to thyroid dysfunction, such as hypothyroidism and subclinical hyperthyroidism. Hypothyroidism occurs in up to 33 % of patients with ectopic thyroid while hyperthyroidism is rare [3, 15]. Our data indicated that hypothyroidism was more frequent than hyperthyroidism in lingual thyroid. One possible reason might be the generally small size of ectopic glands that fail to produce sufficient thyroid hormone, although immaturity and functional deficiency associated with dysgenesis of thyroid glands could not be excluded.

It has been reported that TTF-1 is related to dysgenesis of thyroid gland [18, 19]. Our results indicated that the expression of TTF-1 protein increased in ectopic thyroids compared to orthotopic thyroids. Thus, abnormal expression of TTF-1 is likely to play a role(s) in the occurrence of ectopic thyroid. In addition, elevated TTF-1 expression levels may contribute to the functional abnormality observed in many of ectopic thyroid cases.

TG is exclusively synthesized in the thyroid gland. Our study found that ectopic and orthotopic thyroids were both strongly positive for TG. This indicates that ectopic thyroids are able to carry out thyroid hormone bio-synthesis, even though the quantity of thyroid hormone may be insufficient.

Ki-67-positive tumor cells are often correlated with malignant transformation. There is no previous report regarding Ki-67 expression in ectopic thyroid tissue. This study found that the expression of Ki-67 was low in both ectopic and orthotopic glands, and there was no difference between the two groups. In addition, calcitonin staining was also negative in ectopic thyroid glands. Thus, we did not directly detect signs for malignancy tranformation in ectopic thyroid tissue. However, previous reports showed lingual thyroid carcinoma had an estimated incidence about 1 % [20]. No ectopic thyroid carcinoma was found in our study maybe due to the rarity of the condition.

In humans, the bilateral 3rd and 4th pharyngeal pouches are believed to give rise to parathyroids [21]. Generally, an orthotopic thyroid is usually accompanied with 4 parathyroid glands, but we did not find any parathyroid gland tissue or the expression of PTH in ectopic tissues. Our results may indicate that no accompanying parathyroid could be followed by ectopic thyroid.

## Conclusion

In conclusion, ectopic thyroid is a rare disease. We found that females are more prone to suffer the disease. Lingual thyroid was found to be most common among these patients. Almost half of the ectopic thyroid patients have thyroid hormone deficeincy. Nodular goiter, adenoma, Hashimoto’s thyroiditis had comparable occurrence in ectopic thyroids as in orthotopic thyroid. TTF-1 protein was highly expressed in ectopic tissue, which is likely related to abnormal organogenesis of thyroid glands, leading to its abnormal position and/or functional deficiency. No accompanying parathyroid could be detected with ectopic thyroid. Malignancy was not found in ectopic thyroid tissues in cases with the ectopic thyroids occur between the neck and maxillofacial regions.
